# Development of a Dust Respirator by Improving the Half Mask Frame Design

**DOI:** 10.3390/ijerph18105482

**Published:** 2021-05-20

**Authors:** Oleg Bazaluk, Serhii Cheberiachko, Yurii Cheberiachko, Oleh Deryugin, Vasyl Lozynskyi, Ivan Knysh, Pavlo Saik, Mykola Naumov

**Affiliations:** 1 Belt and Road Initiative Institute for Chinese-European studies (BRIICES), Guangdong University of Petrochemical Technology, Maoming 525000, China; bazaluk@ukr.net; 2 Department of Labour Protection and Civil Safety, Dnipro University of Technology, 49005 Dnipro, Ukraine; sicheb@ukr.net (S.C.); cheberiachkoyi@ukr.net (Y.C.); krempromziz@ukr.net (I.K.); naumov.m.m@nmu.one (M.N.); 3 Department of Transportation Management, Dnipro University of Technology, 49005 Dnipro, Ukraine; oleg.kot@meta.ua; 4 Department of Mining Engineering and Education, Dnipro University of Technology, 49005 Dnipro, Ukraine; saik.nmu@gmail.com

**Keywords:** dust respirator, half mask frame, protective efficiency, anthropometric dimensions, inward leakage coefficient

## Abstract

Protective efficiency of filtering dust respirators depends on the properties of filter materials from which filters are made and the structure of a half mask frame, which influences how tightly the respirator fits the face. The conducted studies on the “Lepestok 40” dust respirator revealed a large air leakage through the gaps occurring along the obturation strip. Therefore, the purpose of the study is to develop a dust respirator to provide high level of protection and usability by improving the half mask frame design. A scheme for designing a dust respirator: analysis of operating conditions for the dust respirator; facial anthropometric measurements of potential users; designing a 3D model of half mask frame; laboratory testing of the protective properties of the product. A distinctive feature of this approach is considering the facial anthropometric dimensions of employees of a particular enterprise, standard sizes formation of 3D facial models, which is the basis for designing a half mask frame for dust respirator. A new half mask frame design for dust respirator with a variable geometry of fitting to the face surface has been developed, due to special attachment points that allow changing its size according to the anthropometric dimensions of user’s face.

## 1. Introduction

Filtering dust respirator (FDR) is the main personal respiratory protective device (PRPD) of a worker from harmful and hazardous substances of the working environment: dust and aerosols. The lack of effective PRPD intensifies the growth of occupational diseases—dust bronchitis and pneumoconiosis, which are most common among workers in the mining industry [1,2,3]. The main reason is the use of PRPDs which do not provide effective protection for the worker when performing production activities [4,5].

Studies on the effectiveness of the FDR application under production conditions of a mining enterprise have revealed the imperfection of the half mask design, which results in leakage of polluted air through the gaps along the obturation strip into the space under mask, which is caused by the peculiarities of the disproportion of the anthropometric dimensions of the user’s face and the size of the respirator half mask [6,7,8,9].

The above-mentioned faults of the FDR design made it possible to form the main objective of the presented study—to develop an efficient FDR to ensure a high level of protection and usability by improving the design of the half mask frame. 

One of the ways to reduce the level of occupational diseases caused by the dust factor is the use of efficient filtering PRPDs. At enterprises of the mining industry, the “Lepestok 40” model has been widely used. This FDR model is currently used at many mining enterprises due to the simplicity of the design and the provision of relatively high protective properties [10,11].

The results of “Lepestok 40” FDR tests which were carried out in the early 2000s to determine compliance with the safety requirements of the European standards contributed to a serious improvement in the design of the model under consideration [12,13]. First, the outer filtering layer was replaced. Secondly, the area of the half mask obturator was extended with an effective cellulose material [14]. Thirdly, the respirator fastening system was changed [15]. Improvements of the FDR received a new name, “SK 200” (“Alina”), successfully completed certification, according to the requirements of the standard [16] as FDR of the second protection class, FFP2.

Research into the practical use of FDR at a mining enterprise revealed the need to develop a more efficient FDR, in whose design a new element has appeared—a frame which makes it possible to completely eliminate the formation of gaps through which polluted air leaks through, due to the possibility of changing the geometry of fitting of the obturator surface to the surface of the employee’s face [17].

## 2. Materials and Methods

**Figure 1:**
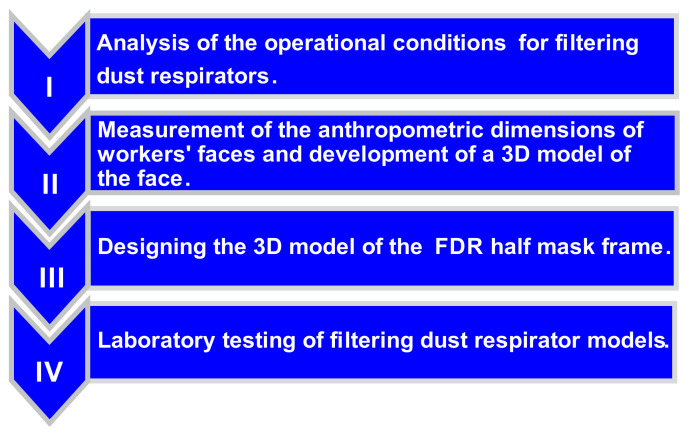
FDR design stages.

The process of manufacturing a modern FDR consists of the following stages (Figure 1).

The first stage includes an analysis of the FDR operating conditions to determine the functional purpose of the protective device, protection class, and selection of appropriate filter materials to manufacture the filter [18]. At this stage, a technical assignment is formed, basic requirements for the structure and materials are written out (Table 1).

At this stage, the main task is solved—to select technical parameters of the FDR filtering materials. Based on the service time, dust holding capacity, working conditions, the size of the fiber diameter, packaging density, the presence of an electrostatic charge on the surface of the fibers, and the thickness and amount of the filter layer are established. For this, well-known dependencies are applied which are used in the Theory of Filtration [19,20,21,22].

(1) Cp=100exp−2β⋅H⋅ηπa,

(2) E=1−Cp=1−exp−2β⋅H⋅ηπa,

(3) $$\Delta p=\mu \cdot v\cdot R_{0}\cdot L,$$

where *C_p_* is the filter protection coefficient; *η* is the aggregate coefficient of particulate matter entrapment; *μ* is dynamic air viscosity, Pa × s; *v* is the filtration rate, m/s; *R*_0_ is dimensionless force of fiber resistance to air flow; *L* is the overall length of filter fibers, m^−1^:

(4) $$L=\frac{\beta \cdot H}{\pi a^{2}},$$

To ensure the required duration of the FDR protective action, their filters are produced being multilayer ones with different packaging density and material thickness. The number of filter layers depends on the operating conditions and, above all, on the air dustiness and the workers’ labour regime [23]:

(5) $$k=\frac{C\cdot Q\cdot t_{k}}{\rho_{p}\cdot V_{\max}},$$

where *k* is the number of filter layers, pcs; *C* is air flow dustiness, mg/m^3^; *Q* is the air-flow rate which depends on complexity of work performance, m^3^/s; *t* is operating time, s; *ρ_p_* is dust particle density, mg/m^3^; *V_max_* is the maximum volume of dust which can be on one filter layer for the specified air flow rate, m^3^.

Elaboration of the design parameters of filtering materials is challenging, for example, the dimensionless force of fiber resistance to the inhaled air flow, which is set experimentally for each filtering material.

**Figure 2:**
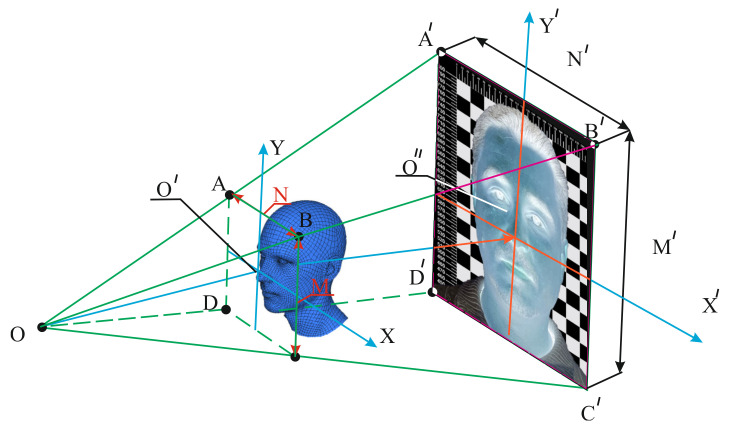
Scheme of a person’s face size recognition.

The second stage includes measurements of the anthropometric dimensions of workers’ faces in order to develop a 3D model of the face; the dimensions are the basis for designing the frames of FDR half masks. Acquisition of anthropometric data on workers’ face sizes for developing or specifying the 3D model dimensions is carried out at a specific coal mining enterprise. In most cases, this procedure is carried out using photographic images which have been taken under certain conditions (with the given resolution, the known distance using a ruler for scaling) (Figure 2).

In the resulting photograph, we determine the contours of the recognition object, including the search for the coordinates of the face in the photograph; we carry out preliminary processing and normalization of critical points; we estimate informative features (main components, local binary patterns, brightness gradient, distances between anthropometric points, etc.) and calculate sizes using key points of anthropometric facial dimensions, which allow determining the head breadth at the forehead, facial width, the face width at the corners of the lower jaw, face length, distance between the eyebrows, head breadth, nose height, nose width near the mouth, nasal bridge width, and nose length.

Next, we form the standard sizes of head models. For this, a well-known approach is used, based on eight basic face sizes, which are fundamental for calculating complex indicators—the so-called PC1 and PC2 components [24,25].

PC1 (the first component) = 0.343264 × (head breadth at the forehead) + 0.426498 × (facial width) + 0.372717 × (face width at the corners of the lower jaw) + 0.329648 × (face length) + 0.363474 × (distance between the eyebrows) + 0.372241 × (head breadth) + 0.113578 × (nose height) + 0.301125 × (nose width near the mouth) + 0.202311 × (nasal bridge width) + 0.193650 × (lip length).

PC2 (the second component) = −0.152951 × (head breadth at the forehead) − 0.039087 × (facial width) − 0.093279 × (facial width, the face width at the corners of the lower jaw or face depth) + 0.359799 × (face length) − 0.173099 × (distance between the eyebrows) + 0.013306 × (head breadth) + 0.551842 × (nose height) − 0.210833 × (nose width near the mouth).

**Figure 3:**
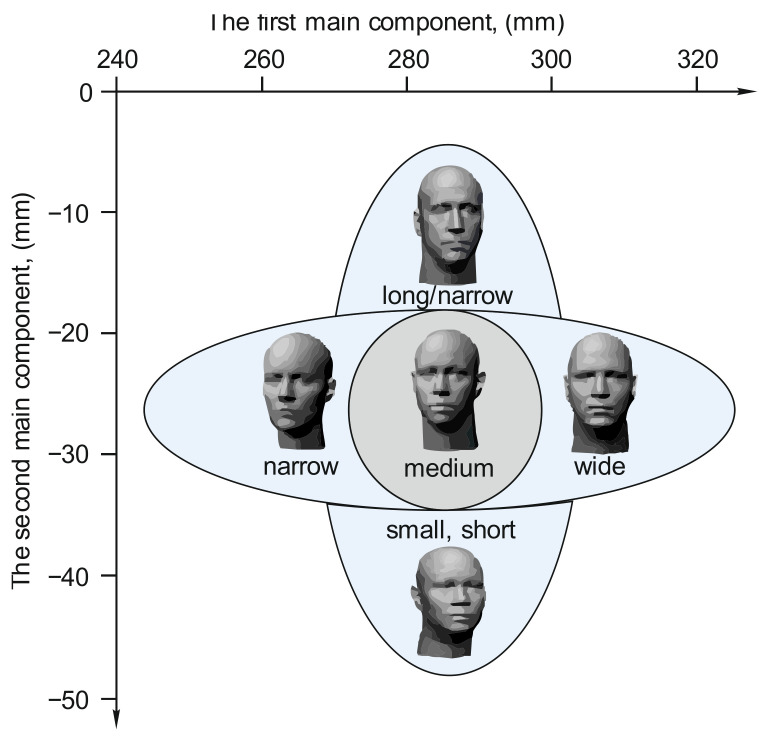
Arrangement of anthropometric sizes of human faces according to the components PC1 and PC2 (adapted from the study [24]).

According to the data of the above components, all types of human faces are divided into five main categories: small, short/wide, medium, long/narrow, large ones (Figure 3), which are then used to form requirements for the FDR design.

The third stage includes designing the frame of the FDR half mask. The transformation of a digital image of a person’s face, given in a three-dimensional coordinate system, into the parameters of an individual FDR half mask is presented in the form of an algorithm [26]:

(6) $$F_{0}=\Pi \left(F_{h}\right),$$

where *F*_0_ is a person’s face shape which has particular values of coordinates *x*, *y*, *z* for points 1, 2, 3, … *n*, which define its parameters in a three-dimensional coordinate system; *F_h_* is an FDR half mask shape conditioned by the change in the original values of coordinates *x*, *y*, *z*; *Π* is the sign of transformation as a result of the design process of the FDR half mask.

**Figure 4:**
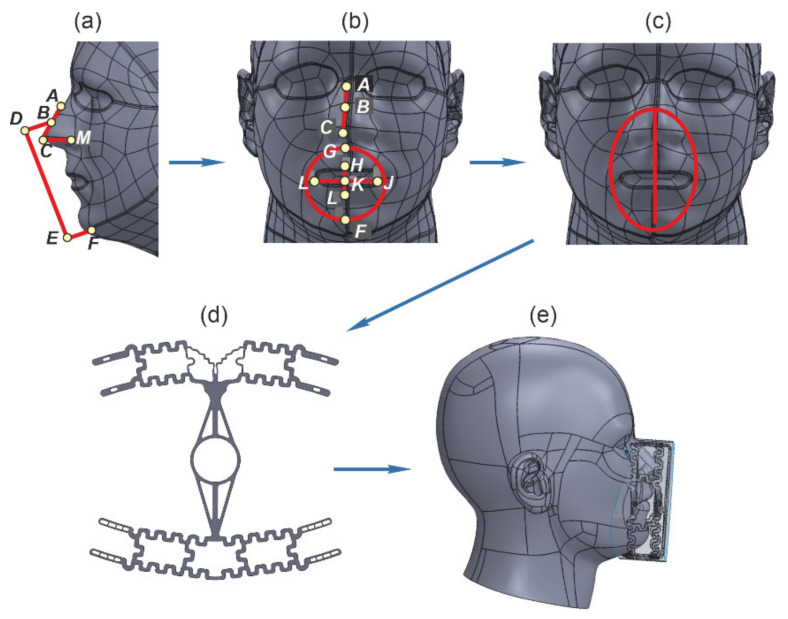
Stages of developing the structure of the FDR half mask frame: (**a**) general view of the head 3D-model; (**b**) contouring the frame; (**c**) frame sketch; (**d**) constructing the frame; (**e**) general view of the frame blank.

The considered algorithm consists of several stages: contouring the FDR half mask along the height and length of the face and determining the shape of the obturation strip; constructing the FDR half mask with the obtained surface of the obturator. This stage is performed with the help of splines, which gradually form the frame of the FDR half mask and connect the main surface elements of the resulting structure (Figure 4).

**Figure 5:**
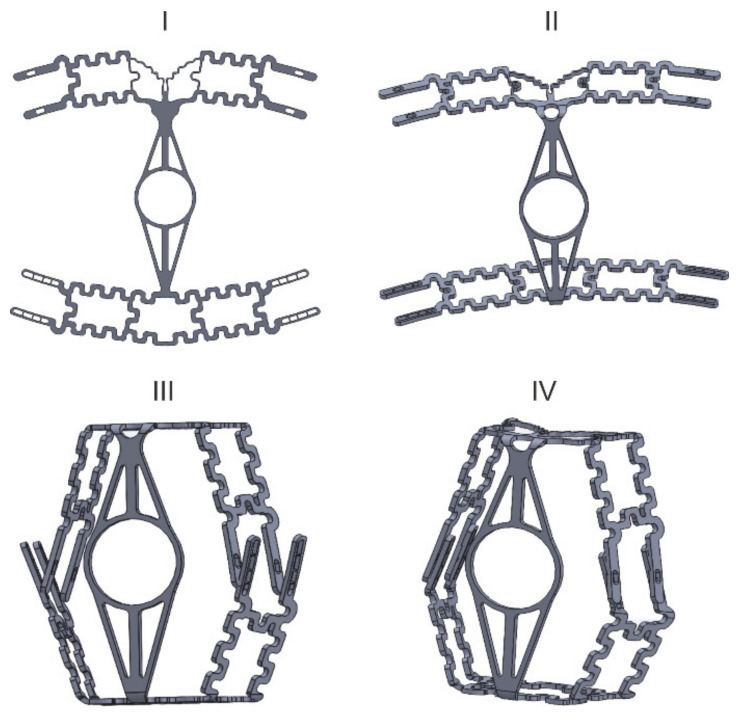
Frame assembly stages: I–IV are an order of elements connecting.

A distinctive feature of the proposed new frame model is the availability of several connecting elements (Figure 5) which allow changing the size of the frame based on the face size together with the location of the cuff around the perimeter of the filter respirator, which together with the obturator forms a channel where the rubber cord is located, whose tightening also contributes to better fitting to the face.

The fourth stage includes manufacturing a model of the FDR half mask and its laboratory testing for compliance with the requirements of the national standards. When manufacturing the FDR frame, it is important to assess its deformation, on which the size of the filtration area depends [27]:

(7) $$S_{filt}=2\pi \cdot (R-u)\cdot (L-u),$$

where *R* is the radius of a spherical segment (half mask), m; *u* is the deformation value, m; *L* is the length of a spherical segment (half mask), m.

The pressure drop Δ*p* is related to the deflection of the shell within the elastic deformations by the dependence [28]:

(8) $$u=\frac{\Delta p\cdot 2E\cdot H}{a^{2}\cdot (1-\xi )},,$$

where *E* is the elasticity modulus of the frame material, Pa; *ξ* is Poison’s ratio.

The modulus of elasticity is set by the type of materials (Table 2) from which the frame can be produced.

As a result of the characteristics analysis of the various materials for printing on a 3D printer [29], the most common polymer ABS-M30i (wire diameter −1.75 mm) was chosen. To manufacture the filters for all respirators, both test and reference, the similar three-layer blanks from polypropylene material “Eleflen” were used (manufactured by the “Standart” Company, Dnipro, Ukraine), with different packaging density of fibers for filtering layers (Table 3): the first layer is protective, made from coarse fibers, the second and third layers are filtering to provide the protection level FFP2.

**Figure 6:**
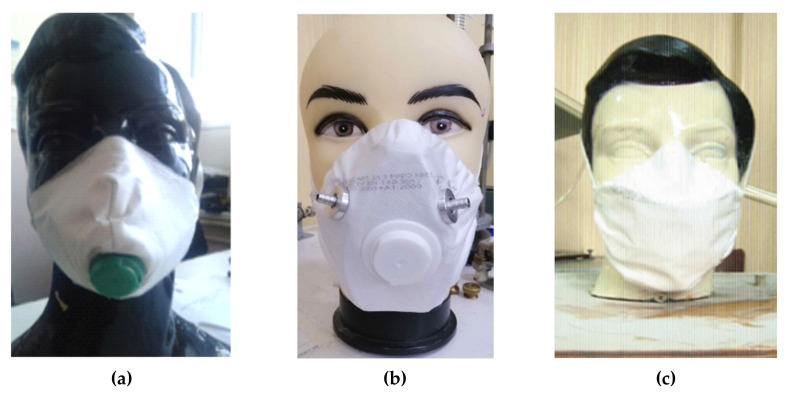
View of the FDR with a frame, with a variable geometry of half mask fitting to the worker’s face: (**a**,**b**) test samples; (**c**) reference sample.

For the analysis, two test samples were manufactured (Figure 6), which differ from each other in the type of the filter attachment to the frame surface.

In option (a), the filter is fastened using the valve saddle, into which the combined mounting holes of the filter itself and the frame are mounted. In this case, fixation is performed by latching in the cage of the protective shield of the valve and the saddle. Additionally, the filter is fastened at the attachment points of the headband, which consists of two sections of elastic band.

In option (b), the filter is fastened along the frame perimeter, by placing a rubber cord along the filter edge (the filter edge is bent to form a channel, welded by ultrasonic welding), which, when tighten together on the inside of the frame, allows the filter to be securely fastened. In this case, the strips are fastened to the filter surface and ultrasonically welded.

## 3. Results

To test the protective efficiency of the developed FDR model with a frame with a variable geometry of the half mask fitting to the worker’s face, laboratory studies on their protective value were carried out with participation of an experimental group for compliance with the international standard requirements [16]. As a reference sample, a filtering dust respirator Standard 203 is used, which has been certified for compliance with the European Council Directive 89/686/EEC on personal protective equipment and meet the requirements of the Technical Regulations for Personal Protective Equipment, approved by the Resolution of the Cabinet of Ministers of Ukraine dated 27 August 2008, No. 761 (https://standart-ua.com/ru/polumaska-filtruyuschaya-standart/ accessed on 13 April 2021).

In the experimental studies, a procedure is presented for determining the penetration coefficient of sodium chloride aerosol into the under-mask space of the developed FDR with the participation of the experimental group. The penetration coefficient was determined as a ratio of the under-mask concentration of the test aerosol to the outer one, which was formed in the test chamber [18,30]:

(9) $$C_{p}=\frac{C_{1}}{C_{2}},$$

where *C*_1_ is the test aerosol concentration in the FDR under-mask space, mg/m^3^; *C*_2_ is the test aerosol concentration in the test chamber, mg/m^3^.

The inward leakage coefficient along the FDR obturation strip was determined as a difference between the penetration coefficient of the respirator as a whole and of the filter:

(10) $$C_{il}=C_{p}-C_{pf},$$

where *C_pf_* is the coefficient of test aerosol penetration through the filter (%), which is defined similarly to *C_p_* by Equation (8).

Subsequently, the respirator protection coefficient was determined by formula [30]:

(11) $$C_{rp}=\frac{100}{C_{p}},$$

For the tests, an experimental group was selected consisting of 10 volunteers with persons with no facial hair-covering (a beard, a moustache, or sideburns). The sample covers a set of characteristics of the anthropometric facial sizes of typical FDR users (Table 4). Before starting to test the filter respirators, a subjective assessment of comfort has been carried out in compliance with the requirements of 8.4 EN 149:2001 + A1:2009, where the testers visually make certain of the structure safety and determine the comfort and reliability of the headband attachment, as well as the limitation of the vision area. As a result, positive reviews have been obtained, which make it possible to draw a corresponding conclusion regarding the satisfactory comfort of the proposed structures. During this test, the headband attachment was damaged in several respirators manufactured according to option (a). These samples were withdrawn and replaced by others.

According to the standard requirements [16], it is imperative to control the anthropometric dimensions of the face: length, width, depth of the face, and length of the lips. To wear the FDR half masks correctly, the experimental group participating in the experiment was introduced to the rules of the correct wearing of the FDR to be tested. If, already in the process of testing, it became necessary to adjust the position of the FDR half mask on the face of a person who took part in the experiment, then the experimental studies were repeated with the restoration of the initial parameters of the system (aerosol concentration, rate of aerosol flow, sampling).

**Figure 7:**
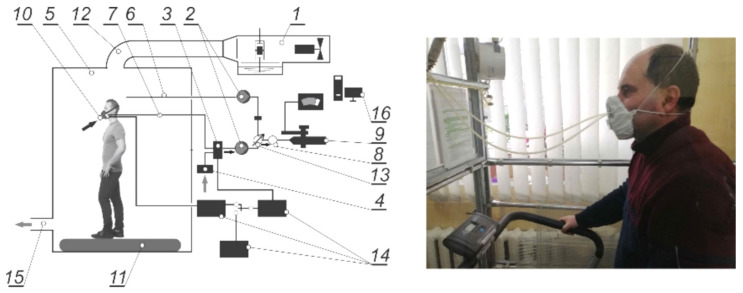
Scheme of a laboratory stand for testing the FDR with test-aerosol with an experimental group participant. Schematic symbols: 1—aerosol generator with a compressor and aerosol line; 2 —aspirator; 3—changeover valve; 4—particulate matter filter; 5—test chamber into which aerosol flows from above; 6—sampling device from the chamber; 7—sampling device from the undermask space; 8—pressure sensor; 9—spectrophotometer; 10—FDR; 11—treadmill located in the test chamber; 12—fresh-air duct and air control valve; 13—clean air inlet; 14—inhalation–exhalation phase distribution system; 15—exhaust ventilation; 16—PC.

The experimental stand, which was used to determine the protective efficiency of the developed FDR with the participation of the experimental group, corresponded to the standard requirements [16]. The general view is shown in Figure 7.

From the test-aerosol generator, the aerosol with a flow rate of 100 dm^3^/min was fed into the test chamber, where its concentration was 8–10 mg/m^3^, while its dispersion was within 0.02–2 µm with an average mass diameter of 0.6 µm. After stabilizing its concentration, its initial concentration in the test chamber was measured using a spectrophotometer. Then, two sampling devices were attached to the front part of the FDR half mask and an air sample was taken from the undermask space with an air flow rate of 15 dm^3^/min to determine the concentration of the test-aerosol in the undermask respiratory space. The measurement results obtained (as they appeared) were automatically entered into a special table, which was created by the AAS-2009 program, where they were statistically processed.

During the experiment, the test person moved on a treadmill at a speed of 6 km/h, and sequentially performed the following movements:

− walking without turning their head or without speaking for 2 min;
− turning their head from side to side (about 15 times) for 2 min (imitation of an overview of the tunnel walls);
− raising and lowering their head up and down (about 15 times) for 2 min (imitation of the view of the floor and ceiling);
− saying the alphabet or another text aloud for 2 min (imitation of a conversation with a colleague);
− walking without turning their head or without speaking for 2 min.

Table 5 presents the results of measuring the FDR inward leakage coefficient for the experimental group participants, and Table 6 shows comparative indicators of the protective efficiency (penetration coefficient, inward leakage coefficient, protection coefficient) of the manufactured FDR model and the “Lepestok 40” half mask.

As a result of the experimental studies, it was found that protective values of the developed FDR half mask meet the requirements of the standard [16] for the FDR half masks of the second protection class. At the same time, the inward leakage coefficient of the proposed design has decreased by over 50% compared to the Standard 203 FDR. The obtained results of the protection coefficient of the considered FDR models depended more on the properties of the filtering material than on the tightness of the half mask fitting to the person’s face. The second protection class is specifically conditioned by application of a polypropylene material for the filter, which, in contrast to the traditional FPP 15–70 material used for manufacturing the “Lepestok 40” FDR, is distinguished by a looser surface of the package and the availability of a surface electrostatic [31,32,33,34,35,36].

## 4. Conclusions

The main reasons that worsen the FDR protective efficiency have been established; these are gaps occurring along the obturation strip, which are formed due to the imperfection of the design of the half mask, obturator, and the head harness fixation.

A design scheme for a FDR half mask has been proposed, which consists of four stages: analysis of working conditions and selection of the necessary filtering material; processing of the anthropometric facial dimensions of potential users; designing a 3D model of the half mask frame; and selection of materials for manufacturing and laboratory testing of protective properties of the finished product.

A new design of the FDR frame with variable geometry has been developed, which allows changing the half mask size in accordance with the anthropometric dimensions of the user’s face.

The experimental tests on the model developed matching requirements of the standard Standard EN 149:2001+A1:2009 shows satisfactory results for the second class of protection, the inward leakage coefficient of the developed respirator design has decreased by more than 50% compared to the “Lepestok 40” FDR model.

## Figures and Tables

**Table 1:** Technical requirements for developing the FDR design (fragment).

Harmful Factor/Type of Activity	Non-Toxic Dust; Low Rate of Work	Non-Toxic Dust; High Air Humidity, High Pace of Work
Operation requirements	Class FFP2	Class FFP3
Design requirements	Availability of an exhalation valve; lightweight filter frame	Availability of inspiratory–expiratory valve assemblies; obturator; filter box for filters
Requirements to materials	Filter material for trapping dust and moisture in the space under mask	Filter material for trapping dust. Insulating (air-tight) materials for the half mask. Plastic for filter boxes. Silicone for inspiratory–expiratory valves.
Benchmarks	Inward leakage coefficient. Penetration coefficient	Inward leakage coefficient. Penetration coefficient. A service time. Breathing resistance

**Table 2:** Characteristics of materials for printing FDR half masks.

3D Printing Material	Melting Temperature, °C	Elasticity Modulus, mPa	Strength Modulus, mPa
ULTEM 1010 ^1^	216	64 (axis XZ) and 42 (axis ZX)	2770 (axis XZ) and 2200 (axis ZX)
PC-ISO ^2^	133	57	2000
Nylon	180	43	1586
ABS-M30i ^3^	96	31	2180
PPSF/PPSF ^4^	230	55	2100
MED610 ^5^	45–50	50–65	75–110
PLA ^6^	49–52	37	1440

^1^ High-performance polyetherimide thermoplastic. ^2^ Biocompatible polycarbonate material. ^3^ Bio-compatible material. ^4^ Polyphenylsulfone. ^5^ Bio-compatible PolyJet photopolymer. ^6^ Polylactic acid.

**Table 3:** Characteristics of material for manufacturing FDR filtering layers.

The Main Parameters of Filtering Layers	Layers		
	One	Two	Three
Mean fiber radius *a*, µm	7–10	2–3	5–7
Fiber packaging density *β*, mg/m^3^	0.06	0.03	0.05
Filter layer thickness, mm	4	3	3

**Table 4:** Anthropometric facial dimensions of the experimental group participating in the experiment.

Anthropometric Facial Dimensions, mm	Test Persons									
	1	2	3	4	5	6	7	8	9	10
Length	111	112	119	120	120	128	127	135	135	136
Width	126	135	125	134	143	124	143	124	136	144
Depth	113	114	100	118	111	109	115	118	114	113

**Table 5:** The experimental value of the inward leakage coefficient when testing the prototype FDR models.

Experimental Group Participant Number	Experimental Value of the Inward Leakage Coefficient				
	When Speaking	Walking	Head Rotation	Head Inclination	Mean Value
1	0.2	0.03	0.04	0.09	0.1
2	0.4	0.2	0.19	0.24	0.33
3	0.45	0.35	0.31	0.4	0.4
4	0.44	0.31	0.31	0.32	0.36
5	1.45	0.98	0.83	0.86	1.03
6	0.7	0.44	0.53	0.37	0.5
7	1.1	0.59	0.63	0.6	0.7
8	0.9	0.79	0.62	0.79	0.8
9	1.2	1.16	1.1	1.1	1.1
10	1.4	1.23	1.2	1.4	1.3
Average	0.82	0.60	0.57	0.61	0.66
Max	1.45	1.23	1.2	1.4	1.3
Min	0.2	0.03	0.04	0.09	0.1

**Table 6:** Experimental group member number of the manufactured FDR model and the Standard 203 half mask.

Identifiable Parameters	Value of Filter Respirator Parameters	
	Standard 203	Prototype Model
Inward leakage coefficient based on test-aerosol sodium chloride, %	1.52 ± 0.08	0.62 ± 0.05
Penetration coefficient based on test-aerosol sodium chloride, %	2.25 ± 0.06	1.15 ± 0.04
Protection coefficient, %	≈44	≈87

## Data Availability

Data are contained within the article.

## References

[1] Nosal D., Konovalov S., Shevchenko V. (2021). Determination of the injury probability among coal mine workers. Min. Min. Depos..

[2] Perederiy G.S., Ponomarenko A.N., Shemyakin G.M., Vetrov S.P. (2009). Occupational risks of industrial dust influence on coalminers in faces of coal miners. Ukr. J. Occup. Health.

[3] Sadovenko I., Ulytsky O., Zahrytsenko A., Boiko K. (2020). Risk assessment of radionuclide contamination spreading while flooding coal mined-out rocks. Min. Min. Depos..

[4] Bazaluk O., Ennan A., Cheberiachko S., Deryugin O., Cheberiachko Y., Saik P., Lozynskyi V., Knysh I. (2021). Research on Regularities of Cyclic Air Motion through a Respirator Filter. Appl. Sci..

[5] Ilyashov M., Diedich I., Nazimko V. (2019). Prospective tendencies of coal mining risk management. Min. Min. Depos..

[6] Kirsch A.A., Budyka A.K., Kirsch V.A. (2008). Filtration of aerosols with fibrous materials FP. Russ. J. Gen. Chem..

[7] Kirillov V.F., Bunchev A.A., Chirkin A.V. (2013). On means of individual respiratory protection of the workers (literature review). Russ. J. Occup. Health Ind. Ecol..

[8] Hoover M.D., Lackey J.R., Vargo G.J. (2001). Independent Evaluation of The Lepestok Filtering Facepiece Respirator. Lovelace Respiratory Research Institute.

[9] Brosseau L.M. (2010). Fit Testing Respirators for Public Health Medical Emergencies. J. Occup. Environ. Hyg..

[10] Denisov E.I. (2014). Masks love counting as well. On the impossibility of reducing hazard classes when using certified PPE for respiratory and hearing organs. Occup. Saf. Health.

[11] Cheberiachko S., Cheberiachko Y., Sotskov V., Tytov O. (2018). National Technical University Dnipro Polytechnic Analysis of the factors influencing the level of professional health and the biological age of miners during underground mining of coal seams. Min. Min. Depos..

[12] Cheberyachko S., Deryugin O., Tretyak O., Pustovoi D. (2019). Revisiting the Improvement of Respiratory System Protection of Mining Workers. Asp. Min. Min. Sci..

[13] Vishnevskaya N.L., Plakhova L.V. (2012). Security issues in the application of respiratory protection devices. Sci. Vector Togliatti State Univ..

[14] Holinko V., Cheberiachko S., Yavorska O., Radchuk D. (2016). Study of protective properties of half-masks respirators used by miners. Min. Min. Depos..

[15] Korobeynikova A.V., Astakhov V.S., Podpletneva G.V. (2005). Use of easy respirators in radiation defense, problems and ways of their solution. Probl. Saf. Nucl. Power Plants Chernobyl..

[16] Standard EN 149:2001+A1:2009. (Main). “Respiratory Protective Devices—Filtering Half Masks to Protect against Particles—Requirements, Testing, Marking”. https://ce-marking.help/directive/personal-protective-equipment/standard/2569/en-1492001-a12009.

[17] Holinko V.I., Naumov N.N., Cheberyachko S.I., Radchuk D.I. (2011). Investigation on the protective efficiency of national disposable dust respirators in accordance with European standards. Metall. Min. Ind..

[18] Koshelev V.E., Tarasov V.I. (2007). On Difficulty of Application of Respiratory Protection in a Simple Way. Perm. Style MG agency. https://www.elibrary.ru/item.asp?id=19540609.

[19] Coffey C.C., Campbell D.L., Zhuang Z. (1999). Simulated workplace performance of N95 respirators. Aiha J..

[20] Ennan A.A., Bolshakov D.A., Gorban L.N. (1999). Weight reduction of Snezhok respirators. J. Weld..

[21] Brown R.C. (1993). An Integrated Approach to the Theory and Applications of Fibrous Filters.

[22] Lin T.H., Chen C.C., Huang S.H., Kuo C.W., Lai C.Y., Lin W.Y. (2017). Filter quality of electret masks in filtering 14.6-594 nm aerosol particles: Effects of five decontamination methods. PLoS ONE.

[23] Sargent E.V., Gallo F. (2003). Use of Personal Protective Equipment for Respiratory Protection. Ilar J..

[24] Lei Z., Yang J., Zhuang Z. (2012). Headform and N95 Filtering Facepiece Respirator Interaction: Contact Pressure Simulation and Validation. J. Occup. Environ. Hyg..

[25] Gumon M. (2017). Respiratory protective devices. Choice and use.

[26] Makowski K., Okrasa M. (2019). Application of 3D scanning and 3D printing for designing and fabricating customized half-mask facepieces: A pilot study. Work.

[27] Menzelintseva N.V., Karapuzova N.Y., Marinina O.N., Stephanenko I.V. (2018). Development of filtering non-woven material for respirators, research and optimization of its properties. Proceedings of higher education institutions. Text. Ind. Technol..

[28] Kornilov O.A. Strength of Materials. Kyiv: «Logos», 2002, 562p. https://www.twirpx.com/file/1003331/.

[29] Melnikova R., Ehrmann A., Finsterbusch K. (2014). 3D printing of textile-based structures by Fused Deposition Modelling (FDM) with different polymer materials. Iop Conf. Ser. Mater. Sci. Eng..

[30] Cheberyachko S., Radchuk D., Yavorska O. (2015). Determination of the Sampling Size for the Filtering Half Mask Laboratory Testing. Metrol. Instrum..

[31] Bbardwaj N., Kundu S. (2010). Electrospinning: A fascinating fiber fabrication technique. Biotechnol. Adv..

[32] Griffin O.G., Longson D.J. (1970). The Hazard Due Inward Leakage of Gas into a Full Face Mask. Ann. Occup. Hyg..

[33] Coffey C.C., Lawrence R.B., Campbell D.L., Zhuang Z., Calvert C.A., Jensen P.A. (2004). Fitting Characteristics of Eighteen N95 Filtering-Facepiece Respirators. J. Occup. Environ. Hyg..

[34] Roberge R.J., Coca A., Williams W.J., Powel J.B., Palmiero A.J. (2010). Physiological impact of the N95 filtering facepiece respirator on healthcare workers. Respir. Care.

[35] Haiko H., Saik P., Lozynskyi V. (2019). The Philosophy of Mining: Historical Aspect and Future Prospect. Philos. Cosmol..

[36] Harber P., Bansal S., Santiago S., Liu D., Yun D., Ng D., Liu Y., Wu S. (2009). Multidomain subjective response to respirator use during simulated work. J. Occup. Environ. Med..

